# Reduced ex vivo susceptibility of *Plasmodium falciparum* after oral artemether–lumefantrine treatment in Mali

**DOI:** 10.1186/s12936-017-1700-8

**Published:** 2017-02-02

**Authors:** Souleymane Dama, Hamidou Niangaly, Amed Ouattara, Issaka Sagara, Sekou Sissoko, Oumar Bila Traore, Amadou Bamadio, Niawanlou Dara, Moussa Djimde, Mohamed Lamine Alhousseini, Siaka Goita, Hamma Maiga, Antoine Dara, Ogobara K. Doumbo, Abdoulaye A. Djimde

**Affiliations:** Malaria Research and Training Center, Faculty of Pharmacy and Faculty of Medicine and Dentistry, University of Sciences, Technique and Technology of Bamako, P.O. Box 1805, Bamako, Mali

**Keywords:** Artemether, Lumefantrine, *Plasmodium*, Ex vivo, In vivo, K13 propeller

## Abstract

**Background:**

Artemisinin-based combination therapy is the recommended first-line treatment for uncomplicated falciparum malaria worldwide. However, recent studies conducted in Mali showed an increased frequency of recurrent parasitaemia following artemether–lumefantrine (AL) treatment.

**Methods:**

Study samples were collected during a large WANECAM study. Ex-vivo *Plasmodium falciparum* sensitivity to artemether and lumefantrine was assessed using the tritiated hypoxanthine-based assay. The prevalence of molecular markers of anti-malarial drug resistance (*pfcrt* K76T, *pfmdr1* N86Y and K13-propeller) were measured by PCR and/or sequencing.

**Results:**

Overall 61 samples were successfully analysed in ex vivo studies. Mean IC_50_s increased significantly between baseline and recurrent parasites for both artemether (1.6 nM vs 3.2 nM, p < 0.001) and lumefantrine (1.4 nM vs 3.4 nM, p = 0.004). Wild type *Pfmdr1* N86 allele was selected after treatment (71 vs 91%, 112 of 158 vs 95 of 105, p < 0.001) but not the wild type *pfcrt* K76 variant (23.5 vs 24.8%, 40 of 170 vs 26 of 105, p = 0.9). Three non-synonymous K13-propeller SNPs (A522C, A578S, and G638R) were found with allele frequencies <2%.

**Conclusion:**

Malian post-AL *P. falciparum* isolates were less susceptible to artemether and lumefantrine than baseline isolates.

## Background

Artemisinin-based combination therapy (ACT) is currently the first-line treatment for uncomplicated falciparum malaria worldwide, in both the public and private sectors [1, 2]. Lumefantrine has never been used alone in people [3]. Studies of in vitro susceptibility of *Plasmodium falciparum* to lumefantrine from West African countries showed IC_50_ ten times below the resistance threshold of 150 nM [4–7], while *P. falciparum* isolates from South-East Asia show higher mean IC_50_ that are closed to that threshold [8].

Several studies have investigated the role of *pfmdr1* gene in lumefantrine tolerance. Sisowath et al. [9, 10] and Baliraine et al. [11] reported that lumefantrine pressure provides the main selective force on the *pfmdr1* N86 allele and leads to resistance to lumefantrine. Happi et al. [12] reported that the *pfmdr1*_ N86_ F184_ D1246 (NFD) haplotype rose from 10% in baseline samples to 75% in breakthrough parasites, an indication of its enhanced survival fitness. Similar observations were reported in three different studies in East Africa [11, 13, 14]. Furthermore, an association between lumefantrine IC_50_ and *pfmdr1* gene polymorphism was demonstrated in Thai isolates [15].

Lumefantrine was shown to have synergistic interaction with artemisinin derivatives in vitro in continuous culture [16]. Hence, artemether was the first artemisinin derivative to be combined in fixed dose with a partner drug as artemether–lumefantrine. Delayed parasite clearance is the phenotype of in vivo resistance to artemisinin and its derivatives in the field [17]. Witkowski et al. [18] reported that increased tolerance to artemisinin in vitro was seen in quiescent *P. falciparum* parasite from Thailand–Cambodia border. Noedl et al. [19] reported the first cases of artemisinin resistance in vitro in Cambodia.

A reduced susceptibility of *P. falciparum* to artemisinin was not detected in mature forms but in young ring stages [20]. The in vitro and ex vivo ring stage survival assay (RSA) on 0–3 h rings (RSA_0–3_) correlated best with in vivo artemisinin resistance as measured by delayed parasite clearance [21]. Flow cytometry-based ring-stage survival assay performs as well as light microscopy and can be used to standardize the collection of RSA data for *P. falciparum* artemisinin resistance between research groups in laboratory and field settings [22].

Recent studies have identified *P. falciparum* Kelch 13 (*pf*K13) polymorphisms to be associated with delayed parasite clearance time after ACT in Southeast Asia [23]. Three key point mutations in the *pf*K13 gene were associated with delayed clearance of *P. falciparum* following artemisinin treatment in Southeast Asia [24]. Most studies assessing the relationship between delayed parasite clearance time and K13 mutations have been conducted in Asia. The possibility that artemisinin resistance might also appear or introduced in sub-Saharan Africa necessitates careful surveillance through periodic in vivo and ex vivo efficacy studies in malaria endemic regions. Although several studies of the efficacy of artemether–lumefantrine in Africa showed high efficacy after molecular correction, the cure rate without PCR correction was lower [25–29]. Similarly, studies in Mali showed a significant number of recurrent parasitaemia following artemether–lumefantrine (AL) treatment [30, 31]. The study hypothesis was that these recurrent parasites are less sensitive to AL in ex vivo study.

## Methods

### Sample collection

Blood samples were collected from the patient enrolled in artemether–lumefantrine arm during the large ongoing network study [32]. Briefly 2–3 ml of blood was obtained by venipuncture from patient with *P. falciparum* mono-infections at least 2250 trophozoites per μl (0.05%) or greater parasitaemia by using the EDTA Vacutainer^®^ tubes. Day 0 samples were collected before treatment administration and independents Day recurrent parasites samples were collected after treatment initiation between Day 28 and Day 42. Samples were transported by car from Sotuba and Kollé to the Culture Room of Malaria Research and Training Centre at 4–8 °C where ex vivo has been done. All samples were cultivated ex vivo immediately or for no longer than 24 h.

### Drugs dilution and plate coating, preparation of culture media

Before sample collection, two anti-malarial drugs (artemether A9361-25MG, SIGMA and lumefantrine L5420-25MG) were dissolved and distributed in appropriate solutions (methanol and ethanol). The starting solutions were prepared from the powder contained in glass vials. Artemether was dissolved at a concentration of 0.1 mg/ml with methanol. Lumefantrine was dissolved in ethanol to have a concentration of 0.01 mg/ml. These starting solutions have been used directly or stored in freezer for the future use. Dilutions calculations were double checked before. All information about weight, solvent were recorded in laboratory book. The preparation of serial dilutions were made in sterile glass flask that allowed to have following concentrations (2.34–600 nM for artemether and 1.25–320 nM for lumefantrine). Each serial concentration have been made in triplicate in 96-well tissue culture plate and dried under a laminar flow hood. RPMI 1640 (R6504-10L) powder with l-glutamate was dissolved in cell grade water and supplemented with sodium bicarbonate and HEPES. After checking pH, which should be 7.2 media was supplemented with 10% of human serum.

### Ex vivo sensitivity testing

For ex vivo drug sensitivity testing, a semi-automated radioactive microplate method was used to measure the IC_50_ as described in a previous study [33]. Briefly, at day 0, ring-form parasites obtained after blood collection were washed three times in RPMI 1640 media. Infected Red blood cells were suspended in 10% human serum completed culture media. After adjustment of parasitaemia between 0.5 and 1% and hematocrit at 1%, an equivalent of 1 μ Ci per well of tritiated hypoxanthine with a specific activity of 14.1 Ci/mmol (Perkin-Elmer, Foster City, CA) was added in a parasite suspension. A final volume of 200 μl per well of parasite suspension was placed into drug-prefilled 96-well tissue culture plates. The parasite suspension was mixed and incubated with each drug at various concentrations of drug at 37 °C with 5% CO_2_, 5% O_2_ and 90% N_2_ for 42 h. After one step of freezing and thawing, the parasites were collected on filter plate, dried and 50 μl of scintillation cocktail (Optiphase Supermix; Perkin-Elmer) was added on each well. Tritium incorporation was determined with a beta-counter (Wallac 1450 microbeta trilux; Perkin-Elmer), and the IC_50_ was determined after the drug concentration was plotted against the radioactivity by the online IC_50_ estimator [34]. Three wells without drug for each concentration panel and the reference clones 3D7 and Dd2 were used as control.

### Molecular analysis

For the molecular analyses purpose dried blood spot samples (DBS) collected during the in vivo study [32]. To evaluate the prevalence of molecular markers of drug resistance and discriminate reinfection from recrudescence, DBS were used to extract parasite DNA using Qiagen mini kit (Qiagen, Valencia, CA) according to the manufacturer’s recommendations.

*Plasmodium falciparum* chloroquine-resistance transporter (*pfcrt*) K76T and *P. falciparum* multidrug resistance 1 (*pfmdr1*) N86Y mutations were genotyped in pre-treatment samples and in parasitaemia occurring in recurrent follow-up day [35]. A nested PCR strategy based on previously published protocols [24, 36] was used to amplify a final 850 bp of the K13-propeller domain product (for PCR conditions see Ref. [36]). PCR products were sent to Macrogen Europe for sequencing. *pf*K13 sequence editing and analysis were conducted in Bamako, Mali.

### Statistical analysis

IC_50_ values were analysed after logarithmic transformation and expressed as the geometric mean of the IC_50_ and the confidence interval 95% (95% CI). These continuous variables were compared using the independent Student’s t test. Prevalence of molecular markers of drug resistance was compared using the Chi Square test. All statistical analyses were done with Stata version 12.0 (Stata Corp., College Station, TX).

## Results

### Ex vivo efficacy

Full in vivo efficacy results for this study were reported elsewhere [32]. Overall 61 samples were successfully analysed in this ex vivo study. 38 samples were collected on day 0 before treatment initiation. Twenty-three samples were collected from recurrent parasites between day 28 to day 42. All samples included in this study had parasitaemia at least 0.05% or greater parasitaemia. 20% of ex vivo samples came from Kollé and 80% came from Sotuba. The geometric mean difference between pre-treatment and post-treatment IC_50_ were 1.6 nM (95% CI, 1.4–1.9) vs 3.2 nM (95% CI, 2.5–4.2) for artemether (p < 0.01) and 1.4 nM (95% CI, 1.2–2.7) vs 3.4 nM (95% CI, 2.0–5.9) for lumefantrine (p = 0.004) (Table 1).

**Table 1:** Geometric mean IC_50_ before and after treatment for artemether and lumefantrine

IC_50_ drugs	Pre-treatment geometric mean (95% ^a^CI)	Post-treatment geometric mean (95% CI)	p values
Artemether	1.6 (1.4–1.9)	3.2 (2.5–4.2)	<0.001
Lumefantrine	1.4 (1.2–1.7)	3.4 (2.0–5.9)	0.004

^a^Confidence interval

### PCR–RFLP analyses of *pfcrt* K76T and *pfmdr1* N86Y single nucleotide polymorphisms

**Fig. 1:**
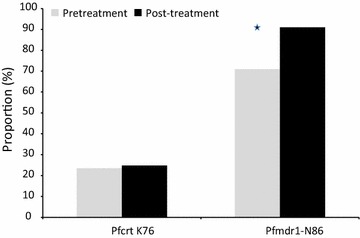
*Pfcrt*K76 and *Pfmdr1*-N86 frequency between pretreatment versus post-treatment parasites (*p < 0.05)

Overall 275 samples were included for the molecular analysis. From which 170 samples were sample available at baseline and 105 were available at the follow-up day. 45% of the sample came from Kolle and 55% came from Sotuba. Prevalence of *pfmdr1* and *pfcrt* alleles were compared between pre-treatment *P. falciparum* parasite isolates and parasites that emerged after treatment. At baseline *pfcrt* K76 was detected in 23.5% (40/170) while *pfmdr1* N86 was detected in 71% (112/158) of *P. falciparum* isolates. After treatment with artemether–lumefantrine the prevalence of *pfcrt* K76 and *pfmdr1* N86 were 24.8% (26/105) and 91% (95/105), respectively (p = 0.9 for *pfcrt* and p < 0.001 for *pfmdr1*) (Fig. 1). There was no statistically significant association between *pfcrt* K76 or *pfmdr*1 N86 and *P. falciparum* ex vivo responses (p > 0.05).

### Kelch13-propeller domain analysis

The K13-propeller domain sequences were generated from 192 samples collected in both Kolle and Sotuba (Genbank accession numbers AD1-AD167). Twenty-five samples had poor quality sequences and were excluded from this analysis. Using 3D7 as the reference strain, three low frequency non-synonymous SNPs were identified at codons A522C (1.2%), A578S (0.6%), and G638R (0.6%) in the K13-propeller domain. Three alleles were observed in Sotuba (3D7, A578S, G638R alleles) and two in Kolle (3D7, S522C allele).

## Discussion

The finding of this study show that the ex vivo efficacy of both artemether and lumefantrine was significantly reduced in post-AL parasites compared to pre-treatment parasites even though the respective IC_50_ were all within the respective sensitive ranges of each drug [37]. The geometric mean IC_50_ values of the artemether measured in the present study are similar to published data from studies conducted in other Africans countries [7, 38, 39]. Artemether mean IC_50_ was six times lower than the 21.2 nM reported in a study conducted in South-Est Asia in 2011 [8]. Lumefantrine mean IC_50_ was lower than that reported in a previous study conducted in Mali [6] and 4 times lower than the 15.1 nM reported in North-Western Thailand [40].

Although there was an increase in IC_50_ in post-AL parasites, in vivo study conducted at the same period of our sample collection found that the corrected clinical efficacy of oral artemether–lumefantrine remained high for the treatment of uncomplicated malaria [32]. All the samples tested ex vivo had an adequate clinical and parasitological response after molecular correction. These results were comparable to previous reports in others sites in Mali [30, 41, 42]. Similar finding was reported in artesunate monotherapy study conducted in Mali in 2010 [25]. The cure rate was also similar to the 99% efficacy reported in a Kenyan study in 2010 [43]. These findings confirm that AL is still efficacious on malaria in Mali.

This study confirms previous findings of a selection of *pfmdr1* N86 following artemether–lumefantrine treatment [9–11, 44]. These molecular findings lend further support to the increased IC_50_ found for lumefantrine as *pfmdr1* N86 was shown to be associated with *P. falciparum* in vitro resistance to lumefantrine [45]. The prevalence of *pfcrt* K76 allele remained stable after treatment with artemether–lumefantrine in this cohort study as reported in recent studies conducted in other Africans countries [46, 47]. The lack of difference of *pfcrt* wild type allele in this dataset between the days of failure compared to the baseline may probably be explained by the high prevalence of the mutant allele at baseline.

The prevalence of K13-propeller mutations was low in Malian parasite population. *In vivo* artemisinin resistance and delayed parasite clearance due to artemisinin resistance have not been reported in Mali [44]. None of the mutations associated with prolonged parasite clearance time after artemisinin-based drugs reported in Cambodia and other Southeast Asian countries were observed in Mali [24]. Of the three non-synonymous SNPs identified in this study only A578S was reported in previous studies in Mali [36, 48]; A522C and G638R have not yet been previously reported. All these SNPs were observed in the pre-treatment samples while no SNPs were observed in post-treatment samples.

The demonstrated continued efficacy of AL in this study has major implications for malaria control in West Africa. Indeed, AL is a centerpiece for malaria case-management in the Sahel belt where current deployment of Seasonal Malaria Chemoprevention (SMC) with sulfadoxine-pyrimethamine (SP) plus amodiaquine (AQ) discourages the use of AQ containing ACTs. In addition, because AL selects the wild type allele *pfmdr*1 N86, which is associated with increased susceptibility to chloroquine and AQ [49], the use of AL in malaria case management in West Africa would prolong the effectiveness of SPAQ for SMC.

Because of logistical constraints complete paired sample set were not obtained for most patients included in the ex vivo assays while paired sample are ideally the best to do before and after. For these reasons independent Student’s t test was used to compare group before and after treatment.

## Conclusion

Although AL is efficacious, ex vivo *P. falciparum* decreased susceptibility and selection *of pfmdr*1 86Y indicate the need not only for aggressive monitoring of parasite response to this mainstream ACT but also for the introduction of new types of ACT in this sub-region.
